# Sir Dyce Duckworth on Prognosis

**Published:** 1896-08-08

**Authors:** 


The Hospital

An Institutional Journal of

The Medical Sciences and Hospital Administration.

with

AN EXTRA NURSING SUPPLEMENT.

Vol. XX.—No. 515.] [Aug. 8, 1896.

### Sir Dyce Duckworth on Prognosis.

If the British Medical Association did nothing else for the profession it would be worth preserving for its annual congresses and for the great opportunities there presented of bringing into prominence those large general principles which constitute medicine a true science and a philosophic art. Without a clear comprehension of those principles the practitioner is a mere handicraftsman and pettifogging trifler with the "rule of thumb." Illuminated and consciously guided by those principles, he is a clear-sighted philosopher and a competent practical guide of suffering humanity in some of the supremest moments of its difficult and much-enduring life. "The trivial round, the common task" in medicine is what we are all too apt to content ourselves with. We need periodically to be forced out of that "trivial round," torn away from that "common task," and made to see our science and the art which is built upon it with the eyes of the philosopher and the man of genius. He it is, and such as he alone, who can give us something like a true measure of the great and infinitely difficult labours we are called upon to perform for a struggling and progressive world.

The prognostician is the physician. The man who cannot prognosticate cannot treat. For prognosis is the summarisation and judicial valuation of the patient and all that appertains to him in his condition as a patient. The man who can prognosticate with judgment can treat with success. The mere names brought together by Sir Dyce Duckworth in the course of his address show the estimation in which prognosis has always been held by the great thinkers of medicine. Hippocrates, Prosper Alpinus, Boerhaave, and in modern times Laycock, Munk, Reynolds, and Allbutt—these have all made a stand in front of prognosis, and have shown their appreciation by definition and exposition, and by an urgent insistence upon the fact that here lies an essential element of all true knowledge and successful practice in our art.

The present generation of practitioners are feeble in prognosis. Such we take to be Sir Dyce Duckworth's judgment in the case. And they are feeble because of their passion for accumulating details. Who of us cannot confirm such a judgment? One of the feeblest of all the prognosticians of the present day—a man well known for his feebleness to many generations of students at a great British medical school—is almost maddening in his dead level repetitions of what seem to be hardly less than millions of details, details which no ordinary human being would have the patience even to enumerate, much less to store in his memory. And this is called "science," nay, more, it goes by the name of "high science." In any other art than that of medicine it would hardly escape the appellation of "imbecility." Sir Dyce Duckworth did well, very well indeed, to expose the futility of the mere accumulation of details in medical practice. Details we must have as a matter of course and by all means. But details without an appreciation of general principles, and the power of measuring and utilising them properly, can no more constitute an art of medicine than heaps of bricks and lime can make a house without the intervention of the architect and the builder.

Semeiology, Sir Dyce Duckworth insists, is the beginning of prognosis; and the most important "semeion" of all is the patient himself. Yet the patient is the very last of all the symptoms and physical signs to be taken into the calculation of the mere man of details. The average "scientist" in medicine never looks at his patient; never thinks of inspecting and "taking stock" of him as a whole. The moment the sick man enters the consulting-room the stethoscope is whipped out, the chest auscultated and percussed, the lungs and heart examined, the liver and spleen investigated, the urine analysed, the facts noted in detail, a prescription written, and the whole thing is done. There was no attempt at the beginning to inspect, no effort at the end to sum up and form a judicial decision. The patient was not a man, not a vital organism; he was a mere conglomeration of rapidly-accumulated details, and was treated accordingly. No wonder it is so often said that the modern physician, with all his science, is less successful in treatment than his professional ancestor, who pretended to no "science" at all!

Where lies the fault in all this? At the medical schools, we are told. The unfortunate medical student, more unhappy than Sisyphus or Tantalus, has about twelve full-blown sciences to face during his hospital career. Every professor of every one of the sciences insists that the dazed youth shall at least know all about his science, whatever happens to the science of the professor in the next room. As the result of all this the untaught, uncultured student comes at last to think that there is no such thing as true medical science, no *bona fide* art of physic with rules and principles to be mastered, but only a certain method of amassing details and prescribing for as many of them as can be brought within the capacity of a physic bottle and six ounces of water. Sir Dyce Duckworth was emphatic that "principles" must be taught at our medical schools; and that the practitioner also must learn to employ "principles." "Those of us," he insisted, "who have had long and chastening experience of practice both in public and private" have realised the imperative necessity of a clear comprehension of each case that is submitted to us as a whole, and of dealing with the case as a living, thinking, and feeling organism, who is to be made or marred by our judgment and skill. Thinking capacity is the great essential of all the endowments of the practical physician; and compared with this the mere power to accumulate details is a futility and an exasperating pedantry.

